# Extent of maxillary deficiency in patients with complete UCLP and BCLP

**DOI:** 10.1186/1746-160X-10-26

**Published:** 2014-06-20

**Authors:** Jörg A Lisson, Catharina Weyrich

**Affiliations:** 1 Department of Orthodontics, University Hospital and Dental Medical School Saarland, Homburg/Saar, Germany; 2 Klinik für Kieferorthopädie (Geb. 56), Universitätsklinikum des Saarlandes, Kirrberger Str. 100, 66424 Homburg/Saar, Germany

**Keywords:** Le fort I-osteotomy, UCLP and BCLP patients, Maxillary deficiency, Inhibition of craniofacial growth

## Abstract

**Objectives:**

Primary surgery in patients with complete unilateral and bilateral cleft lip and palate restricts transverse and sagittal maxillary growth. Additional surgical maxillary advancement might become necessary after completion of growth. The aim of this study was to determine the extent of maxillary deficiency at an early stage during the transitory dentition, and to identify factors that might indicate the need for a later maxillary advancement.

**Materials and methods:**

Lateral head films and casts of 40 non-syndromatic patients with complete UCLP (n = 29) and BCLP (n = 11) were evaluated. This retrospective evaluation included measurements of casts and lateral head films from all patients at the beginning of orthodontic treatment during the transitory dentition (T1), after completion of orthodontic treatment (T2) and after completion of growth (T3). The statistic analysis comprised t-tests (Anova) and correlation analyses (Pearson).

**Results:**

SNA decreased significantly between T1 and T2. At T3, 27.5% of the patients showed a sagittal maxillary deficiency with need for osteotomy. There were no statistical differences between patients with UCLP and BCLP. Significant positive correlations occurred between SNA and WITS-appraisal (+0.62), and significant negative correlations between SNA and NL/NS (−0.66).

**Conclusions:**

During craniofacial growth patients with complete UCLP and BCLP experience sagittal growth inhibition of the maxilla after primary surgery. A later need for maxillary advancement after completion of growth occurs equally in both cleft types. There are no correlations regarding the need for osteotomy with gender or number of primary surgical measures. It is impossible to predict a need for later maxillary osteotomy during the transitory dentition.

**Clinical relevance:**

Patients with clefts typically receive long-term treatment. The present results provide useful information for treatment planning and implementation.

## Introduction

Treatment of patients with facial clefts starts after birth and ought to be finished after the completion of craniofacial growth. It is always mandatory to apply treatment through an interdisciplinary team if optimal functional and esthetic results are desired. In any case, the treatment regimen includes surgical closure of lip, alveolus and palate in patients with a complete cleft. Despite atraumatic procedures are applied, midfacial growth restriction will occur due to scar tissue [1-4], leading to orthodontic treatment need at a later stage.

Two factors might lead to unfeasible results. The hereditary component in patients with a skeletal Class III anomaly showing clefts can hardly be addressed by orthodontics alone. Still, even without this factor dentofacial orthopedic and orthodontic treatment is often not capable of compensating maxillary skeletal discrepancies resulting from scar-induced growth impairment, thus leading to unfeasible functional and esthetic results [5-8]. These patients show a need for maxillary advancement after completion of growth [7]. However, several studies reveal a great variety for the need for maxillary advancement [5,7,9-17]. These differences can be explained with the heterogeneity of the different studies.

Ross [7] investigated a large number of males (n = 100) with complete unilateral cleft, but the patients received their individual treatment from different surgeons and with different treatment protocols. The common denominator was the cleft, ignoring confounding factors deriving from these. Ross concluded that 27% of the patients received an additional maxillary advancement after completion of growth. Other studies even included patients with incomplete or isolated cleft of lip or palate [12,17]. A Le-Fort-I-osteotomy became necessary in only in 20.9% of the patients [12]. However, extracting patients with complete uni- or bilateral cleft of lip, alveolus and palate from this study reveal a necessity for maxillary advancement in 47.7% of the investigated persons.

The need for additional maxillary surgical advancement in case of maxillary deficiency is obviously given when tooth movement alone cannot correct a present malocclusion. Limitations of tooth movement are patient age and the extension of the alveolar process. Proffit [18] has described the “envelope of discrepancy” that shows the possible extent of tooth movement with and without additional osteotomy of upper and/or lower jaw. Still, it is difficult for the practitioner to determine a definite threshold between a conservative and a surgical approach during treatment of borderline cases. If the wrong decision is made in the process of treatment, its length will greatly increase due to the necessity to switch from compensation to decompensation with consecutive surgery. The disadvantages for the patient are obvious: increased treatment time, risks and costs.

This retrospective study in patients with unilateral and bilateral complete cleft, lip and palate intends to determine indicators for the decision in favor of or against additional surgical maxillary advancement.

## Methods

The study was conducted according to the Helsinki Declaration. Anonymized data from 40 non-syndromatic patients with complete unilateral (n = 29) and bilateral (n = 11) cleft, lip and palate were evaluated. All patients received their entire rehabilitation according to the treatment protocol applied at Saarland Medical School, Homburg, Germany (Table 1). Homburg is the only location to provide complete cleft care in Saarland, a comparatively small state with a population of approximately one million (400 per km^2^). All patients were at least 17 years old.

**Table 1:** Procedures for surgical and orthodontic treatment at Saarland Medical School, Homburg, Germany

**Treatment time**	**Procedure**
0 - 12 months	Presurgical orthopedics
6 months	Lip closure after Veau-Manchester, if possible along with hard palate closure after Pichler
10-12 months	Soft palate closure after Veau with push-back procedures after Kriens
Primary and transitory dentition	Orthodontic treatment according to individual needs (e.g. frontal or lateral cross bite)
Before eruption of the permanent canine	Bone grafting
Secondary dentition	Orthodontic treatment (fixed appliances)
16 years or after	Cosmetic corrections of nose and lip,
	LeFort-I-Osteotomy

This retrospective evaluation included casts and lateral head films taken from all patients at the beginning of orthodontic treatment during the transitory dentition (T1), after completion of orthodontic treatment (T2) and after the completion of growth (T3). The mean age at T1 was 10.30 ± 1.52 years, at T2 14.83 ± 1.11 years and at T3 17.82 ± 1.58 years. No patient received additional surgery apart from primary procedures until T3.

Cast measurements were performed using a caliper (Beerendonk, Dentaurum 042–750, Pforzheim, Germany) with an accuracy of 0.1 mm and included the parameters

• Overjet and overbite (mm)

• Anterior and posterior arch width (mm)

Lateral head films were evaluated using OnyxCeph (version 2.7.19, Image Instruments, Chemnitz, Germany) and included the parameters

• SNA [°]

• SNB [°]

• ANB [°]

• Wits-Appraisal [mm]

• NL/NSL [°]

• ML/NSL [°]

• ML/NL [°]

• Ui/NL [°]

• Li/ML [°]

These measurements are widely used, therefore comparison to results of other studies is ensured.

Since the definition of a sagittally deficient maxilla is only vaguely described as retroposition or retrusion, more criteria had to be used to gain a more thorough description of deficiency. In this study, a maxilla was considered deficient if 3 out of 4 criteria were fulfilled:

• Overjet < −2 mm with Li/ML within means

• SNA decreasing at least 4° below means

• Ui/NL increasing at least 3° above means with Li/ML within means

• Wits-Appraisal < −1.5 mm with SNB within means

The statistic analysis was performed using SPSS 19.0 (SPSS, Chicago, ILL, USA) and comprised means and standard deviations. Regarding their normal distribution, the continuous variables were tested using the Kolmogorov-Smirnov-test. A normal distribution could be calculated for nearly all variables. Thus, tests for normally distributed samples were applied for the comparison of the samples. Means were then compared using paired t-tests for intra-group comparisons. Pearson’s correlation test was applied to the variables.

The error of measurement was calculated by re-evaluation of five sets of lateral head films and casts by the same experienced investigator after a twelve-week interval using Dahlberg’s formula. [19]. Cast measurements showed mean errors of 0.16 mm, cephalometric values presented mean errors of 0.62° for angular and 0.61 mm for linear measurements.

## Results

The cast measurements revealed a significant decrease of the overjet between T1-T3 and T2-T3 (p < .05). The overbite showed no significant changes (p > .05). The anterior arch width increased significantly between T1-T3 (p < .01), while the posterior arch width did not change remarkably. All cast results are presented in Table 2.

**Table 2:** Changes of cast analysis

**Measurement**	**Time of treatment**	**Δ Mean**	**SD**	**p-value**	**Significance**
	Δ T1-T2	0.11	2.43	0.769	n.s.
Overjet [mm]	Δ T2-T3	−0.95	1.91	0.010	*
	Δ T1-T3	−0.85	2.26	0.040	*
	Δ T1-T2	−0.60	2.18	0.096	n.s.
Overbite [mm]	Δ T2-T3	−0.19	1.72	0.553	n.s.
	Δ T1-T3	−0.83	2.64	0.095	n.s.
	Δ T1-T2	1.069	3.84	0.910	n.s.
Anterior arch width [mm]	Δ T2-T3	0.276	2.94	0.611	n.s.
	Δ T1-T3	1.321	3.71	0.042	*
	Δ T1-T2	0.687	4.37	0.333	n.s.
Posterior arch width [mm]	Δ T2-T3	0.033	2.56	0.084	n.s.
	Δ T1-T3	0.732	3.94	0.310	n.s.

(T1: age 10 years; T2 age 15 years; T3: age 18 years; n.s.: not significant; *: significant (p < 0.05).

Cephalometrics showed significant changes for sagittal parameters (Table 3). SNA decreased between T1-T2 (p < .001) and between T1-T3 (p = .003). SNB increased between T1-T3 (p = .001). ANB decreased accordingly both between T1-T2 and T1-T3 (p < .001). The same changes could be observed for the WITS-Appraisal between T1-T2 and T1-T3 (p < .001). Vertical parameters underwent no significant changes apart from an increase of ML/NSL (p = .018). A highly significant protrusion of the upper incisors (Ui/NL, p < .001) could also be observed between T1-T3.

**Table 3:** Changes of cephalometric analysis

**Measurement**	**Treatment interval**	**Δ Mean**	**SD**	**p-value**	**Significance**
	**Δ** T1-T2	−2.57	3.40	0.000	***
SNA [°]	Δ T2-T3	−0.53	3.54	0.490	n.s.
	Δ T1-T3	−2.76	3.98	0.003	**
	Δ T1-T2	0.91	2.60	0.034	*
SNB [°]	Δ T2-T3	0.95	1.93	0.030	*
	Δ T1-T3	2.18	2.85	0.001	**
	Δ T1-T2	−3.49	2.46	0.000	***
ANB [°]	Δ T2-T3	−1.39	3.08	0.046	*
	Δ T1-T3	−4.85	3.29	0.000	***
	Δ T1-T2	−3.48	3.85	0.000	***
Wits [mm]	Δ T2-T3	−1.05	3.24	0.162	n.s.
	Δ T1-T3	−4.89	3.67	0.000	***
	Δ T1-T2	−0.17	3.37	0.745	n.s.
NL/NSL [°]	Δ T2-T3	−0.55	3.18	0.424	n.s.
	Δ T1-T3	−0.74	4.43	0.430	n.s.
	Δ T1-T2	−0.40	3.34	0.457	n.s.
ML/NSL [°]	Δ T2-T3	−1.01	2.22	0.050	n.s.
	Δ T1-T3	−2.03	3.70	0.018	*
	Δ T1-T2	−0.47	4.12	0.480	n.s.
ML/NL [°]	Δ T2-T3	−0.29	3.53	0.706	n.s.
	Δ T1-T3	−0.81	4.44	0.103	n.s.
	Δ T1-T2	10.56	9.99	0.000	***
Ui/NL [°]	Δ T2-T3	2.26	6.77	0.131	n.s.
	Δ T1-T3	11.41	11.20	0.000	***

(T1: age 10 years; T2 age 15 years; T3: age 18 years; n.s.: not significant; *: significant (p < 0.05); **: highly significant (p < 0.01); ***: most highly significant (p < 0.001)).

The correlation between SNA and posterior arch width (OK66) remained unchanged (p > .05), the same accounts for the correlation between SNA and Ui/NL. Significantly positive correlations were found between SNA and WITS-Appraisal at T1 (p = .01), at T2 (p < .001) and T3 (p = .003). Further positive correlations occurred between SNA and anterior arch width (OK44) at T2 (p = .039) and T3 (p < .025). The correlation between SNA and NL/NSL was significantly negative at all times (T1 and T2 p < .001, T3 p = .020). All results of the Pearson-Correlation are presented in Table 4.

**Table 4:** Pearson-Correlation

**Measurement**	**Correlation coefficent**	**p-value**	**Significance**
SNA-Wits T1	0.447	0.010	*
SNA-Wits T2	0.619	0.000	***
SNA-Wits T3	0.598	0.003	**
SNA-OK44 T1	0.108	0.509	n.s.
SNA-OK44 T2	0.336	0.039	*
SNA-OK44 T3	0.500	0.025	*
SNA-OK66 T1	−0.040	0.809	n.s.
SNA-OK66 T2	0.276	0.093	n.s.
SNA-OK66 T3	−0.024	0.920	n.s.
SNA-UI/NL T1	−0.279	0.081	n.s.
SNA-UI/NL T2	−0.305	0.059	n.s.
SNA-UI/NL T3	−0.165	0.451	n.s.
SNA-NL/NSL T1	−0.599	0.000	***
SNA-NL/NSL T2	−0.663	0.000	***
SNA-NL/NSL T3	−0.481	0.020	*

(OK44: anterior arch width; OK66: posterior arch width; (T1: age 10 years; T2 age 15 years; T3: age 18 years; n.s.: not significant; *: significant (p < 0.05); **: highly significant (p < 0.01); ***: most highly significant (p < 0.001)).

The chi-square testing revealed no significant correlations (p > .05) between maxillary deficiency and the total number of surgical procedures, the gender or the cleft type (Table 5).

**Table 5:** Chi-Square-Test according to Pearson

**Measurement**	**Chi-square test**	**p-value**	**Significance**
Number of operations – Maxillary deficiency	4.649	0.098	n.s.
Gender – Maxillary deficiency	0.101	0.751	n.s.
Type of cleft – Maxillary deficiency	0.482	0.786	n.s.

(n.s.: not significant).

At T3, 27.5% of the patients showed a maxillary deficiency (Table 6). 17.5% of the patients were in need of surgical maxillary advancement. A maxillary deficiency was already present at T1 in 2.5% and at T2 in 12.5% of the patients.

**Table 6:** Number of patients (absolute and percentage) with maxillary deficiency at T1, T2 and T3 and patients with LeFort-I-Osteotomy after completion of growth

**Measurement**	**Number of entities**	**Percentage [%]**
Patients with maxillary deficiency (T1)	1/40	2.50
Patients with maxillary deficiency (T2)	5/38	13.20
Patients with maxillary deficiency (T3)	11/40	27.50
Patients with LeFort-I-Osteotomy	7/40	17.50

## Discussion

The present study investigated casts and lateral cephalograms of 40 non-syndromatic patients born between 1975 and 1993 with complete unilateral or bilateral cleft lip and palate. The surgical and orthodontic treatment had always followed the same concept. The patients showed the same distribution of 3:1 between unilateral and bilateral cleft as described in other studies [20]. The same counts for the localization of unilateral clefts, which occur preferably on the left side (59%).

Despite all measurements were performed twice by the same observer using the same tools, errors of the measurements occur. This cannot be avoided since even the same observer defines anatomical landmarks differently at varying time points, thus leading to inconsistencies of measurements [21].

The evaluation of the casts revealed a continuous decrease of the overjet from 0.75 ± 2.93 to 0.19 ± 2.65 between T1 and T3. 24% of the patients showed a reverse overjet. Capelozza et al. [22] investigated the overjet in patients with complete cleft in groups with and without surgery. The mean overjet always increased in patients without surgery, whereas it always decreased in patients with surgery. The explanation for this phenomenon is seen in the scar tissue of the lip and scar traction at the palate occurring after surgery [7,22]. Maulina et al. [23] could show that a reverse overjet may be avoided through early orthodontic treatment during which the overjet is corrected successfully.

The overbite also decreased between T1 (2.50 ± 2.45 mm) and T2 (1.70 ± 1.55 mm). Although not present in the investigated group, this reveals a tendency towards anterior open bite in patients with complete cleft lip and palate. Vertical growth impairment appears to be as present as the sagittal restriction during growth and treatment [4].

The anterior arch width is initially small compared to the posterior at T1. After palate closure, lateral segments show torsion towards the midline [3,24] which explains this initial finding. During the observation period, the anterior arch width increased which is a result of the administered orthodontic treatment.

SNA decreased between T1, T2 and T3, resulting in mean 77.63 ± 4.94°. Trotman and Ross [25] found the same results in a group of patients with complete bilateral cleft lip and palate between 6 and 12 years and as grown-ups. They also observed a decrease of SNA as consequence of surgical procedures, since SNA undergoes a physiologic increase during growth in non-cleft patients [26].

SNB always increased significantly. According to the findings of Björk and Skjeller [27], this can be interpreted as normal growth.

The changes of ANB did occur accordingly. Treutlein et al. [28] investigated 10 year old patients with complete unilateral cleft. Their findings were analogous to those of this study at T1. If ANB shows a mean 4.67 ± 3.77° this can be interpreted as average with a tendency towards mandibular retrognathia. At T1, the pubertal growth spurt had most likely not occurred. At T3, ANB was only 0.29 ± 3.39°, thus allowing the interpretation that maxillary growth impairment sets in during puberty until completion of growth.

The same course occurs for the values of the WITS-Appraisal. Being positive at T1, the mean values continuously decrease and become negative until T3. While the mean values for SNB represented normal behavior of the mandible, the mean changes of the WITS-Appraisal also indicates maxillary growth impairment between T1 and T3.

Vertical measurements revealed a tendency towards clockwise rotation of maxilla and mandible. This has been previously described for the maxilla [4,24,29] and appears to be a characteristic of patients after surgical closure of a complete cleft. Some authors also described this for the mandible [22,30]. The present results show a significant decrease of ML/NSL between T1 and T3. During the transitory dentition, a slight clockwise rotation of the mandible can be observed but is no longer present at T3. Lisson and Tränkmann [2] presented the same effect for patients with complete bilateral cleft lip and palate but without need for osteotomy.

Incisor inclination in the present study at T1 is comparable to values found in the literature: patients with operated cleft lip and palate show retroinclined upper incisors when compared to non-cleft individuals [24,31], probably as a result of increased lip tension after surgery. During orthodontic treatment between T1 and T3 the upper incisors became markedly protruded. The resulting Ui/NL mean value of 109.92 ± 9.18° can be interpreted as dentoalveolar compensation of a skeletal maxillary deficiency.

A positive correlation between SNA and WITS could be observed at all times, the results are according to those of Lisson et al. [32].

A highly significant correlation exists between SNA and NL/NSL. The negative correlation between these angles explains the connection of decreasing SNA and a clockwise rotation of the maxilla [17,24].

However, a positive correlation between the formation of a maxillary deficiency and the number of surgical interventions could, unlike in the literature [12], not be found. Patients with unilateral complete cleft lip and palate show the necessity for maxillary osteotomy just as often as those with bilateral complete cleft. There are no gender-specific differences. Good et al. [12] arrived at different conclusions and described a connection between cleft type, number of surgical interventions and growth restriction in the maxilla.

27.5% of the patients in this study showed a deficient maxilla resulting in the need for additional osteotomy. 11 of these patients (17.5%) actually received a Le-Fort-I-osteotomy. The differentiation between maxillary deficiency and actually performed Le-Fort-I-osteotomy allows a better differentiation than other studies, in which the need for additional surgery is determined only in patients who already underwent surgery [9,12]. Not every patient in need of maxillary advancement actually decides in favor for it. Since this aspect is completely missing in other studies, the presented figures must be interpreted carefully. The comparison of the present results with those of the literature shows that the values for absolute need for osteotomy are in the lower range (27% in [19], 47.4% in [12], 48.3% in [9]), indicating the usefulness of the applied interdisciplinary treatment approach.

At T1 during the transitory dentition, only one out of 40 patients (2.5%) showed a maxillary deficiency. This patient was also one of those with the need for maxillary advancement after therapy. No other patient showed signs for a later need for LeFort-I-osteotomy at T1. Therefore, it appears that a prognosis of a later need for osteotomy is rather difficult during the transitory dentition. It seems possible to conclude that mandibular forward growth [27] with simultaneous deficient maxillary forward [25] growth during the pubertal growth spurt may be responsible. More information about the individual growth type ought to be gained from further head films at a later stage. Studies like this would greatly benefit from larger patient numbers, but migration of patients often prohibits successful follow-up investigations.

## Conclusion

During craniofacial growth, patients with complete unilateral and bilateral cleft of lip, alveolus and palate experience characteristic growth inhibition of the maxilla after completion of primary surgery. A later need for surgical maxillary advancement after completion of growth occurs equally in both cleft types. There are no correlations regarding the need for osteotomy with gender or number of primary surgical measures. It is impossible to predict a need for later maxillary osteotomy during the transitory dentition. However, every patient with a cleft shows an individual hereditary growth pattern, which again may cause significantly different treatment needs.

## Competing interests

The authors declare that they have competing interests.

## Authors’ contributions

JAL: Conceptualized and designed the study, structured the initial draft of the manuscript, critically reviewed the manuscript, and approved the final manuscript as submitted. CW: Performed research and collected data, and drafted the initial manuscript. All authors read and approved the final manuscript.
